# Predictors of Visual Acuity Outcomes after Anti–Vascular Endothelial Growth Factor Treatment for Macular Edema Secondary to Central Retinal Vein Occlusion

**DOI:** 10.1016/j.oret.2021.02.008

**Published:** 2021-11

**Authors:** Piyali Sen, Sarega Gurudas, Jayashree Ramu, Namritha Patrao, Shruti Chandra, Rajna Rasheed, Luke Nicholson, Tunde Peto, Sobha Sivaprasad, Philip Hykin

**Affiliations:** 1 NIHR Moorfields Biomedical Research Centre, London, United Kingdom; 2 UCL Institute of Ophthalmology, London, United Kingdom; 3 NetwORC UK Reading Centre, Queen’s University of Belfast, Belfast, United Kingdom

**Keywords:** LEAVO, BCVA, VEGF, Predictors, BCVA, best-corrected visual acuity, CI, confidence interval, CRVO, central retinal vein occlusion, CST, central subfield thickness, ELM, external limiting membrane, ERM, epiretinal membrane, EZ, ellipsoid zone, fp, fractional polynomials, LR, likelihood ratio, ME, macular edema, OR, odds ratio, SD, standard deviation, VA, visual acuity, VEGF, vascular endothelial growth factor

## Abstract

**Purpose:**

To evaluate whether baseline demographic, clinical, and OCT characteristics predict visual acuity (VA) outcomes in patients receiving anti–vascular endothelial growth factor (VEGF) therapy for macular edema (ME) due to central retinal vein occlusion (CRVO).

**Design:**

Post hoc analysis of the randomized noninferiority trial (Lucentis, Eylea, Avastin in CRVO) LEAVO Study from December 12, 2014, to December 16, 2016, carried out across 44 UK National Health Service ophthalmology departments.

**Participants:**

Data on 267 participants with a baseline best-corrected mean visual acuity (BCVA) range of 19 to 78 Early Treatment Diabetic Retinopathy Study letter score (approximate Snellen equivalent, 20/32 to 20/320) who had central subfield thickness (CST) ≥ 320 μm on Spectralis OCT (Heidelberg Engineering) were analyzed.

**Methods:**

Study participants were randomized to receive repeated intravitreal injections of ranibizumab (0.5 mg/50 μl), aflibercept (2.0 mg/50 μl), or bevacizumab (1.25 mg/50 μl), and a protocol-driven pro re nata re-treatment regimen at 4 to 8 weekly visits was followed up to week 100 after 4 mandated 4-weekly loading injections.

**Main Outcome Measures:**

Change in BCVA and percentage of patients gaining ≥ 10 letters and achieving BCVA letter score > 70 letters at 52 and 100 weeks.

**Results:**

The analysis was adjusted for treatment effects and confirmed by sensitivity analysis. Age ≥ 75 years is a poor predictor for all 3 visual outcomes. Lower baseline BCVA predicted 10-letter gainers and higher gains in BCVA, although it is a poor predictor of achieving > 70 Early Treatment Diabetic Retinopathy Study letters. None of the baseline OCT morphologic characteristics except ellipsoid zone (EZ) integrity influenced any visual outcomes. Both baseline CST and total macular volume showed a nonlinear relation to 10-letter gainers, with CST > 900 μm being a poor prognostic indicator. Baseline CST and macular volume did not predict mean change in BCVA or BCVA > 70 letters at 52 and 100 weeks. The sensitivity analysis conclusions after removing iCRVO were similar.

**Conclusions:**

At presentation, younger age, higher baseline BCVA, and a definitely intact subfoveal EZ are predictors of BCVA score > 70 letters at 100 weeks.

Macular edema (ME) is the major cause of visual impairment in patients with central retinal vein occlusion (CRVO).^1^ OCT is the key imaging modality for diagnosis and monitoring ME treatment response.^2^ Furthermore, re-treatment criteria are based on visual acuity (VA) and OCT central subfield thickness (CST) changes from baseline or previous visit. Therefore, we evaluated whether visual prognosis after treatment with anti–vascular endothelial growth factor (VEGF) therapy for ME secondary to CRVO could be predicted by baseline VA and OCT parameters.

Several reports emphasize the association of certain ME morphologic features with poor VA, including the presence of macular subretinal fluid, loss of integrity of outer retinal layers including the ellipsoid zone (EZ) or external limiting membrane (ELM), disorganization of the inner retinal layers, hyper-reflective foci, large cystoid spaces, and vitreoretinal interface abnormalities.^3^, ^4^, ^5^, ^6^ These parameters have been evaluated principally in diabetic ME.^7^ However, in CRVO, the onset of ME is typically more acute and initial macular volume and CST are greater than in diabetic ME. Therefore, we evaluated whether there are CRVO-specific OCT predictors of visual outcome.

Previous reports have only studied short-term outcomes of best-corrected visual acuity (BCVA) predictors in CRVO-related ME.^8^^,^^9^ This report describes analyses of visual outcomes in the LEAVO participants based on baseline VA and Spectralis OCT (Heidelberg Engineering) parameters to better understand treatment benefit by 100 weeks. The 52-week outcome is reported as a secondary outcome. The LEAVO study is a randomized, controlled, prospective, multicenter, noninferiority trial that compared 3 available anti-VEGF agents for ME due to CRVO.^10^ At week 100, the primary outcome showed bevacizumab was not inferior, whereas aflibercept was not inferior to ranibizumab. The proportion of participants who gained ≥ 10 letters at 100 weeks was not statistically different between treatment arms (ranibizumab 63%, aflibercept 68%, and bevacizumab 63%). Only participants who had Spectralis OCT throughout the study timelines were included because key grading features are better visualized with this device.

## Methods

The LEAVO study was conducted across 44 clinical sites throughout the United Kingdom. Each patient provided informed consent, and the study was ethics approved (National Research Ethics Service Committee London - London Bridge, 04/09/2014, ref: 14/LO/1043). The study adhered to the Declaration of Helsinki.

### Participants

Eligible patients for this study had baseline BCVA Early Treatment Diabetic Retinopathy Study letters between 19 and 78 and spectral domain OCT CST ≥ 320 μm due to ME secondary to CRVO of less than 12 months duration in the study eye. Key exclusion criteria included ME due to other ocular pathology including diabetic retinopathy, any eye condition affecting VA during the study, or intravitreal injection of corticosteroids 90 days or anti-VEGF therapy 60 days before recruitment were excluded. A study participant could have only 1 study eye.^11^

### Treatment Regimen

After 4-weekly anti-VEGF injections until week 12, week 16 and 20 visits were mandated where treatment was given at these and all subsequent visits to week 96 only if re-treatment criteria were met. Re-treatment criteria included a decrease in VA of ≥ 6 letters due to increase in CST or an improvement in VA ≥ 6 letters between the current and most recent visit, or CST > 320 μm or > 50 μm increase in CST from lowest recorded reading.

### Assessment

Standardized refraction was performed by certified optometrists and Early Treatment Diabetic Retinopathy Study BCVA letter score measured at 4 m. Postmydriatic Spectralis OCT images were obtained by certified operators. Retinal morphology was assessed using the Spectralis macular raster with dimensions of 30 × 25 degrees and 31-line scans at 241-μm spacing. Five horizontal OCT scans at baseline, 1 B-scan encompassing the fovea, and 2-line scans covering 500 μm superior and 500 μm inferior to the fovea, respectively, were assessed for morphologic features as defined in Table S1 (available at www.ophthalmologyretina.org/). All scans were analyzed by masked graders at the NetwORC UK Reading Centre and morphologic features by masked and trained retinal fellows at Moorfields Eye Hospital. The anti-VEGF agent and number of injections received by each patient were recorded.

### Outcomes

The VA outcomes included change in BCVA at 100 weeks, proportion of 10-letter gainers (final BCVA baseline ≥ 10 letters), and BCVA > 70 letters at 100 weeks.

### Statistical Analysis

Baseline characteristics of the study population are summarized using mean (standard deviation [SD]) or n (%). Initially, analysis was conducted on the complete sample of observations available at the univariate level to retain as many samples as possible for statistical power (available-case analysis). Furthermore, covariates with less than 5% missing or ungradable were set to missing and excluded from the analysis; otherwise, where there were more than 5% ungradable observations, these were modeled as a separate category or missing indicator.^12^ Correlations between BCVA and the continuous parameters at baseline and week 100 were investigated using Spearman’s rank correlation coefficient (95% confidence interval [CI]). The baseline characteristics and OCT parameters were analyzed using linear and logistic regression for the continuous and binary outcomes, respectively. Continuous variables were modeled using fractional polynomials (fp),^13^^,^^14^ where a linear fit was not adequate. The fp transformations were described using component-plus-residual plots. Likelihood ratio (LR) tests and Akaike information criterion were used to compare linear and nonlinear models. Linear splines were also generated and plotted to verify nonlinearity in situations where there might be potential multicollinearity between fp terms. All analyses were adjusted for treatment type, age, baseline VA, and disease duration. Variables with *P* values > 0.1 in the univariate-adjusted analysis qualified for inclusion in the multivariable models where backward elimination was performed with a stay criterion set to a nominal *P* value of 5%. Explanatory power of the final models was interpreted using the R^2^ index (Mcfaddens R^2^ for logistic regression models). Analysis was repeated at week 52. In the sensitivity analysis, because of a small proportion of participants presenting with ischemic CRVO at baseline, we replicated all analysis after excluding these patients. The selection criteria for the optimal fp functional form (alpha) was increased from 0.05 to 0.1 because of reduced sample size. The nonlinear functional form was also verified using flexible linear splines. All statistical analysis was performed in Stata version 16.^15^

## Results

A total of 267 of 463 randomized participants in the LEAVO trial had Spectralis OCT data and completed the 2-year visit (Fig S1 shows the participant flowchart, available at www.ophthalmologyretina.org/). In this study cohort, 28 participants (10.49%) had ischemic CRVO at baseline. The treatment allocation for this cohort were ranibizumab (n = 92), aflibercept (n = 89), and bevacizumab (n = 86). Table 1 shows the baseline characteristics of these participants and the treatment received.

**Table 1:** Baseline Characteristics and Treatment Received

Characteristics	Total (n = 267)
Clinical Characteristics at Baseline and Treatment over 100 Wks	
Age, mean (SD), yrs	68.6 (13.3)
< 50 yrs (n %)	22 (8.2%)
50–74 yrs (n %)	145 (54.3%)
≥ 75 yrs (n %)	100 (37.5%)
Female (n %)	115 (43.1%)
Duration of CRVO at Diagnosis	
Median (IQR), mos	0.93 (0.37–1.83)
< 1 mo	143 (53.6%)
≥ 1 mo	124 (46.4%)
Baseline BCVA, mean (SD)∗	53.8 (15.1)
> 70 letters	28 (10.5%)
55–70 letters	130 (48.7%)
37–54 letters	64 (24.0%)
< 37 letters	45 (16.9%)
Anti-VEGF Agents (Total Participants)	
Ranibizumab	92 (34.5%)
Aflibercept	89 (33.3%)
Bevacizumab	86 (32.2%)
Mean No. of Injections (SD) by 100 Wks	
Ranibizumab	12.8 (5.0)
Aflibercept	10.2 (3.8)
Bevacizumab	12.5 (5.4)
OCT Parameters at Baseline	
Baseline CST, mean (SD)†	723.1 (226.9)
Total volume, mean (SD)‡	12.96 (2.93)
Proportion with ME N (%)	
Ungradable	1 (0.4%)
Diffuse only	22 (8.2%)
Cystoid only	208 (77.9%)
Mixed	36 (13.5%)
Size of Largest Cyst in Cystoid/Mixed Edema (n = 244)	
Small (< 250 mm)	43/244 (17.6%)
Medium (≥ 250– < 500 mm)	148/244 (60.6%)
Large (≥ 500 mm)	53/244 (21.7%)
Proportion with Subretinal Fluid, N (%)	
Absent	85 (31.8%)
Present	169 (63.3%)
Ungradable	13 (4.9%)
Proportion with VMIA (ERM or VMT) N (%)	
No evidence	233 (94.33%)
Present	14 (5.67%)
Ungradable	20 (7.50%)
Ellipsoid Zone, N (%)	
Intact	87 (32.6%)
Not intact	41 (34.8%)
Ungradable	139 (52.06%)
DRIL, N (%)	
Absent	144 (53.9%)
Presence	118 (44.2%)
Ungradable	5 (1.9%)
External Limiting Membrane, N (%)	
Intact	112 (42.0%)
Not intact	24 (9.0%)
Ungradable	131 (49.1%)
Hyper-reflective Foci N (%)	
Absent	158 (59.2%)
Present	109 (40.8%)
Ungradable	0

BCVA = best-corrected visual acuity; CRVO = central retinal vein occlusion; CST = central subfield thickness; DRIL = disorganization of retinal inner layers; ELM = external limiting membrane; ERM = epiretinal membrane; EZ = ellipsoid zone; ME = macular edema; SD = standard deviation; SRD = subretinal detachment; VEGF = vascular endothelial growth factor; VMIA = vitreomacular interface abnormalities; VMT = vitreomacular traction.

∗ The BCVA of 4 participants was set to missing because they did not meet eligibility criteria or did not complete 4-m tests despite having baseline BCVA ≤ 19/data entry error from the site.

† The CST data were missing for 3 participants at screening.

‡ Total volume data were missing for 5 participants at screening.

### Correlations

**Figure 1:**
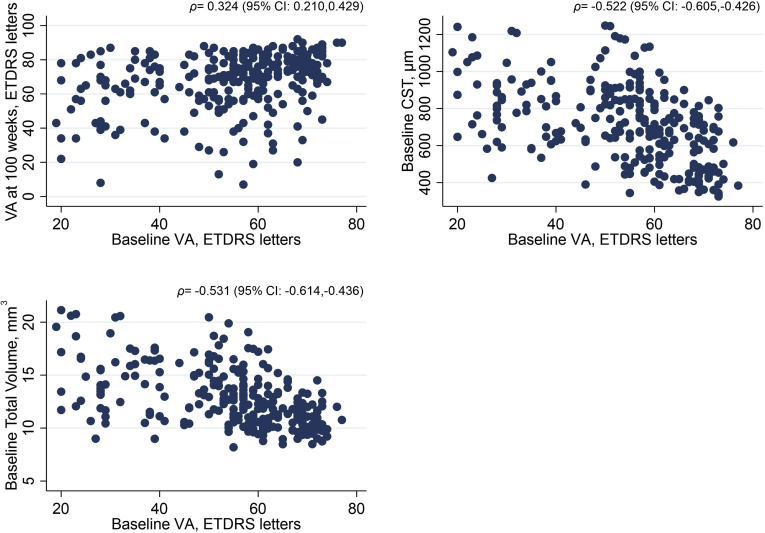
Correlation plots for BCVA, CST, and total macular volume. Scatter plots to show relationships among continuous variables BCVA, CST, and total volume. The 95% confidence interval (CI) for the Spearman’s correlation coefficient is based on Fisher’s transformation. BCVA = best-corrected visual acuity; CST = central subfield thickness; ETDRS = Early Treatment Diabetic Retinopathy Study; ρ = spearman’s rank correlation coefficient; VA = visual acuity.

Figure 1 shows the Spearman’s correlation coefficients (95% CI) between baseline BCVA with CST, total macular volume, change in BCVA at 100 weeks, and final BCVA at 100 weeks. Table S2 (available at www.ophthalmologyretina.org/) shows the correlations between baseline risk factors in the primary cohort and testing associations between all baseline risk factors. Baseline BCVA correlated with all OCT variables except subretinal detachment. Those with ungradable or questionable EZ and ELM had a higher than average CST (ELM: mean, 823.6 μm; SD, 173.2 and EZ: mean, 807.4 μm; SD, 180.1), compared with CST in patients with these layers being intact at baseline (ELM: mean, 574 μm; SD, 167.5, EZ: mean, 539 μm; SD, 144). These differences were statistically significant (Table S3, available at www.ophthalmologyretina.org/).

### Relationship among Baseline Demographics, BCVA, and VA Outcomes

Results from adjusted analyses restricted to demography, baseline BCVA, and ocular characteristics from screening visit OCT are shown in Table 2. Eyes that presented with lower BCVA at baseline were more likely to see larger gains in final BCVA (both by mean change and improving by 10 or more letters) but less likely to reach a score > 70 letters by 100 weeks. After adjusting for injection type, every 10-letter increase in baseline BCVA was associated with a 3.5 (95% CI, 2.1–4.9) letter increase in absolute BCVA at 100 weeks (*P <* 0.001), 42% reduction in the odds of improving by ≥ 10 letters (95% CI, 28–54), and 56% increase in the odds of reaching > 70 letters at 100 weeks (95% CI, 29–89). Further adjusting for age and disease duration did not greatly affect the magnitude or precision of the effect of 3.6 (95% CI, 2.3–5.0) letter decrease in final BCVA; 44% (95% CI, 29–55) reduction in odds of improving by ≥ 10 letters; and 62% (95% CI, 32–97) increase in odds of reaching > 70 letters at 100 weeks. Likewise, younger age was associated with a larger magnitude of treatment benefit on BCVA at 100 weeks (every year of older age associated with mean VA gains of −0.33 [95% CI, −0.48 to −0.19] at 100 weeks) in the adjusted analysis. Sex was not associated with VA outcomes at 100 weeks. The adjusted R^2^ for change in VA from baseline to 100 weeks was 15.2% for a model adjusting for these identified baseline factors (age, disease duration, baseline VA, and treatment arm). For the binary outcomes, adjusted Mcfadden’s R^2^ was 7.4% and 6.7%, corresponding to 10-letter gainers and those reaching VA of 70 or more by 100 weeks.

**Table 2:** Visual Acuity Outcomes at 100 Weeks, by Demographic Variables, Baseline BCVA, and OCT Characteristics

Patient Characteristics	Final BCVA at Week 100∗		BCVA Improvement ≥ 10 Letters		Final BCVA > 70 Letters	
	Estimate (95% CI)	*P* Value	OR (95% CI)	*P* Value	OR (95% CI)	*P* Value
Demography and Baseline VA						
Age†∗∗††						
< 50	Ref	−	Ref	−	Ref	−
50–74	−5.59 (−12.84–1.66)	0.13	0.78 (0.25–2.43)	0.67	0.24 (0.07–0.75)	*0.01*
≥ 75	−13.09 (−20.57–−5.61)	*0.001*	0.38 (0.12–1.20)	0.10	0.15 (0.04–0.48)	*0.001*
Age†∗∗††‡‡ (linear)	−0.33 (−0.48–−0.19)	*< 0.001*	0.97 (0.94–0.99)	*0.004*	0.96 (0.94–0.98)	*0.001*
Disease duration†	−1.06 (−2.06–−0.07)	*0.04*	0.90 (0.78–1.02)	0.11	0.89 (0.78–1.02)	0.10
Sex†						
Male	Ref	−	Ref	−	Ref	−
Female	−1.59 (−5.59–2.42)	0.44	0.98 (0.57–1.66)	0.93	1.08 (0.64–1.80)	0.78
BCVA, Letters †∗∗††‡‡						
> 70	Ref	−	Ref	*−*	Ref	−
55–70	−6.96 (−13.69–−0.24)	0.04	2.03 (0.87–4.73)	0.10	0.65 (0.26–1.62)	0.36
37–54	−12.76 (−20.03–−5.50)	*0.001*	4.84 (1.99–11.76)‡	*0.001*	0.24 (0.09–0.63)	*0.004*
< 37	−17.58 (−25.50–−9.65)	*< 0.001*	−		0.14 (0.05–0.41)	*< 0.001*
BCVA †∗∗††‡‡ (linear)	0.35 (0.21–0.49)	*< 0.001*	0.95 (0.93–0.97)	*< 0.001*	1.05 (1.03–1.07)	*< 0.001*
OCT Characteristics§						
CST, μm‖‡‡				*0.003*		
Linear/fp terms	−0.01 (−0.02–0.004)	0.18	2 FP terms¶	*0.001*	1.00 (0.999–1.002)	0.91
Volume, mm^**3**^‖‡‡	−0.18 (−1.00–0.64)	0.66	2 FP terms#	*0.004* *0.002*	1.02 (0.91–1.15)	0.70
SRD‖						
Absence	Ref	−	Ref	−	Ref	−
Presence	−1.36 (−5.48–2.77)	0.52	0.86 (0.47–1.58)	0.64	1.04 (0.58–1.85)	0.80
DRIL‖						
Absent	Ref	−	Ref	−	Ref	−
Present	−0.59 (−4.78–3.60)	0.78	0.98 (0.53–1.82)	0.96	1.06 (0.58–1.93)	0.85
EZ‖∗∗††‡‡						
Intact	Ref	−	Ref	−	Ref	−
Not intact	−15.90 (−21.47–−10.33)	*< 0.001*	0.18 (0.07–0.47)	*< 0.001*	0.20 (0.07–0.56)	*0.002*
Ungradable/questionable	1.34 (−3.02–5.69)	0.55	1.85 (0.94–3.62)	0.08	2.26 (1.14–4.48)	0.02
ELM‖∗∗‡‡						
Intact	Ref	*−*	Ref	−	Ref	–
Not intact	−10.47 (−17.36–−3.58)	*0.003*	0.32 (0.11–0.92)	*0.04*	0.57 (0.20–1.63)	0.29
Ungradable/questionable	2.98 (−1.24–7.20)	0.17	1.65 (0.89–3.06)	0.11	2.04 (1.10–3.78)	0.02

AIC = Akaike Information Criterion; BCVA = best-corrected visual acuity; CI = confidence interval; CST = central subfield thickness; DRIL = disorganization of retinal inner layers; ELM = external limiting membrane; EZ = ellipsoid zone; fp = fractional polynomial; LR = likelihood-ratio test; OR = odds ratio; SD = standard deviation; SRD = subretinal detachment; VA = visual acuity.

Statistically significant *P* values at the 5% threshold (*P <* 0.05) are italicized.

Fractional polynomial terms:

¶ CST: Term 1: Xˆ2–50.69 and Term 2: Xˆ3–360.89; fp model AIC = 302 versus linear model AIC = 314.

# Total volume: Term 1 Xˆ2–1.65, Term 2: Xˆ3–2.12; fp model AIC = 306 versus linear model AIC = 313. The LR test comparing linear and nonlinear models (*P <* 0.001; LR chi-square = 13.22 for CST and *P =* 0.002; LR chi-square = 9.75 for total volume from an LR test). Model AIC is interpreted as an out-of-sample prediction error and can used to compare nested models. Variables that remained significant after adjustment for total injection number indicated with:

∗∗ (continuous BCVA),

†† (final VA > 70 letters), and

‡‡ (BCVA improvement ≥ 10 letters).

∗ For baseline VA, the outcome should be interpreted as the final VA at 100 weeks.

† Adjusted for baseline VA and treatment arm.

‡ Groups 37–54 and < 37 were collapsed for outcome because of low numbers in group < 37 letters that did not improve. One bivariate outlier was identified in VA change (67 letters decrease) after truncating at 3 SD, and 3 further outliers were identified and removed from CST and total volume.

§ Showing only variables that were statistically significant at the 10% threshold (*P <* 0.1).

‖ Adjusted for baseline VA, age, disease duration, and treatment arm.

### Relationship between Baseline OCT Characteristics and VA Outcomes

**Figure 2:**
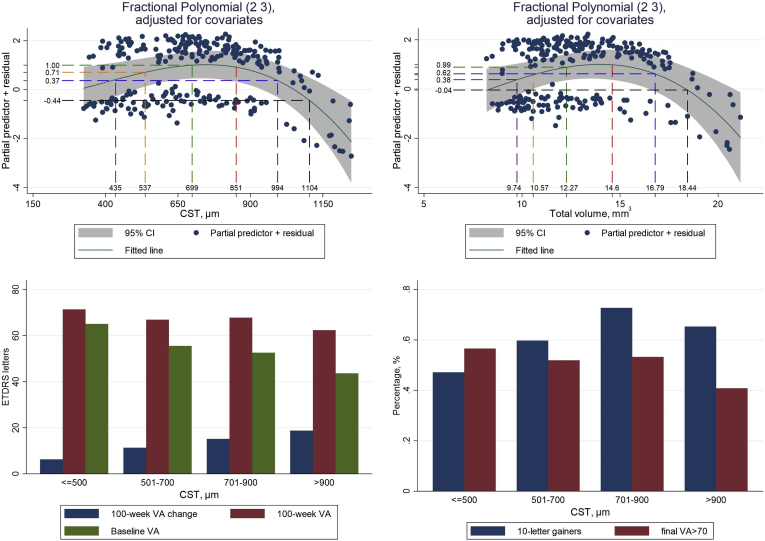
Effect of CST and total macular volume on visual acuity (VA) outcomes. All (2 3) fractional polynomial (fp) terms represent xˆ2 and xˆ3 transformations. **Top:** Component plus residual plots for fp models for CST and total volume adjusting for baseline factors age, disease duration, baseline BCVA, and treatment arm. The x-axis represents the range of values across which the continuous variable was observed, and y-axis shows the log odds for predicting an improvement in VA of ≥ 10 letters at 100 weeks. The smooth line represents the fitted curve, the shaded line represents the 95% CI for the fit, and the points represent the residuals. The lines indicate the 10^th^ (purple), 25^th^ (orange), 50^th^ (green), 75^th^ (red), 90^th^ (blue), and 95^th^ (black) percentiles. **Bottom:** Bar plots for different vision outcomes across 4 subgroups of CST for (a) change in VA, final VA, and baseline VA and (b) the proportion of 10-letter gainers and those reaching > 70 letters final VA. BCVA = best-corrected visual acuity; CI = confidence interval; CST = central subfield thickness; ETDRS = Early Treatment Diabetic Retinopathy Study; VA = visual acuity.

After adjusting for baseline BCVA, age, disease duration, and treatment type, only EZ and ELM were found to be significantly associated with the outcomes (Table 2). The CST and total macular volume both exhibited nonlinear relationships with the log-odds of 10-letter gain (Fig 2). Both variables were better modeled using fp over their linear counterparts, which provided an inadequate model fit (*P <* 0.001 and *P =* 0.002; LR test for CST and total volume, respectively). As expected, both variables showed similar effect relationships due to high intercorrelation (ρ = 0.867; 95% CI, 0.833–0.895). Variables were subsequently remodeled using linear splines, recovering a similar explanatory model (Fig S2, available at www.ophthalmologyretina.org/). Furthermore, despite added complexity introduced by the fp terms, model Akaike information criterion remained lower than the linear model. Central subfield thickness of approximately 750 μm indicated maximum log-odds of BCVA improvement of ≥ 10 letters, thereafter seeing a decline in log-odds. On average, CST > 900 μm showed a 66% reduction in the odds of improving by ≥ 10 letters compared with those between 700 and 900 μm (odds ratio [OR], 0.34; 95% CI, 0.14–0.83, *P =* 0.018). The concavity of the curves (initial increase) may be explained by the intercorrelation between baseline BCVA and CST. For instance, eyes with CST that is marginally greater than 320 μm are subject to a ceiling effect of baseline BCVA (Fig 2, bar plot), whereas extremely thick CST is less likely to improve because of the extremity of the CST and therefore poor prognosis. A similar effect was seen in total macular volume, where a value of 8.96 mm^3^ (lower 2.5th percentile) had an average predicted probability of 54%, increasing to 73% at 14 mm^3^, indicating maximum odds of improving. Values of total volume > 14 mm^3^ showed a steady decline in the probability of improving ≥ 10 letters, decreasing to 28% for an individual with total volume of 19.9 mm^3^ (upper 2.5th percentile).

### Multivariable Analysis

**Figure 3:**
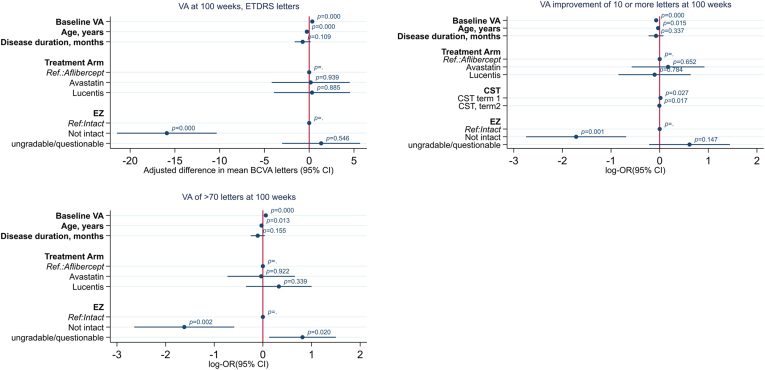
Forest plots from multivariable analysis of outcomes at week 100. Variables that passed the *P <* 0.1 threshold in the univariate (adjusted) analysis were subsequently included in the multivariable models (external limiting membrane [ELM], ellipsoid zone [EZ], and CST). Backward elimination was carried out locking in control variables or confounders for precision regardless of statistical significance and setting the variable elimination threshold at *P <* 0.05. In all models, ELM was eliminated at the 5% level (not presented). BCVA = best-corrected visual acuity; CI = confidence interval; CST = central subfield thickness; ETDRS = Early Treatment Diabetic Retinopathy Study; VA = visual acuity.

Variables EZ, ELM, CST, and total macular volume qualified to enter the multivariable model for predicting a gain of ≥ 10 letters; however, after baseline adjustment of the demographic features and treatment, ELM subsequently decreased. Figure 3 summarizes all variables that remained in the final multivariable models. For participants with nonintact EZ, the mean BCVA gains were 15.9 letters less, and 10-letter gains (OR, 0.18; 95% CI, 0.07–0.47) or achieving 70 letters by 100 weeks were less likely (OR, 0.57; 95% CI, 0.2–1.63). For 10-letter gainers after adjusting for EZ, CST and total volume were modeled using fp functions (Fig S3, available at www.ophthalmologyretina.org/). Age and baseline BCVA remained significantly associated in all models, but in this study disease duration was not found to be independently related to VA when considering the OCT parameters. In this model, adjusted R^2^ for the change in BCVA over 2 years was 27.8% (vs. 15% for a model adjusting for demographics, baseline VA, and drug assignment only). For 10-letter gainers, McFadden’s adjusted R^2^ increased to 17.4% (vs. 7.4%). For predicting final VA > 70 letters, McFadden’s adjusted R^2^ increased to 13.2% (vs. 6.7%), where values 20% to 40% indicate good to excellent fit.^16^

### Week 52 Analysis

For the analysis at week 52, demographic variables and baseline BCVA exhibited similar relationships as week 100 analyses (Table S4, available at www.ophthalmologyretina.org/), in which age, disease duration, and baseline VA remained statistically associated with the outcomes after adjusting for treatment arm and baseline VA. Nonlinearity was detected for the continuous variable CST for predicting a gain in BCVA of ≥ 10 letters (Fig S4, available at www.ophthalmologyretina.org/), but were not retained in the multivariable model. Total volume did not exhibit a statistically significant linear relationship with the outcome, and no fp power led to an improvement in the model fit (LR test, *P* > 0.05). *P* values for EZ and ELM remained statistically significant in the adjusted analysis for the outcome pertaining to BCVA at 52 weeks (Table S5, available at www.ophthalmologyretina.org/). Only EZ was related to the outcome of improving by ≥ 10 letters, and no morphologic parameter showed a statistically significant association with the outcome of reaching 70 letters or more by week 52 despite adjustment for baseline BCVA, treatment arm, age, and disease duration. Results from the multivariable analysis showing the ocular characteristics indicative of good treatment response are presented in Figure S5 (available at www.ophthalmologyretina.org/).

### Sensitivity Analysis

Table S6 (available at www.ophthalmologyretina.org/) shows the data separately for participants with ischemic versus nonischemic CRVO at baseline. Participants on average had a lower baseline VA, higher baseline CST, higher total volume, and greater gains in BCVA at 100 weeks. By week 52, the proportion achieving greater than 70 letters was higher in the ischemic group (57%) than in the nonischemic group (50%), whereas by 100 weeks a higher proportion was seen in those with nonischemic CRVO at baseline (52% vs. 43%). The proportion gaining 10 or more letters was consistently higher in the ischemic group at both 52 weeks (74%) and 100 weeks (78%). Table S7 (available at www.ophthalmologyretina.org/) shows the adjusted analysis restricted to demography, baseline BCVA, and ocular characteristics from screening visit OCT against 100-week VA outcomes after excluding participants with ischemic CRVO at baseline. All variables that were statistically significant from the analysis on the total cohort remain significant at the 1% level despite the reduced sample size. The fp terms (Fig S6, available at www.ophthalmologyretina.org/) indicate a similar functional form as observed in the total cohort for the 10-letter gainers at 100 weeks. The nonlinear model compared with the model with linear terms for CST and total volume had a significantly better model fit (*P <* 0.05; LR test for both CST and total volume). Likewise, the analysis using linear splines at 100 weeks (Fig S7, available at www.ophthalmologyretina.org/) shows a similar association for both CST and total volume (*P <* 0.05; LR test against linear model for both CST and total volume). Table S8 (available at www.ophthalmologyretina.org/) shows the analysis at week 52 and indicates that the associations for OCT parameters remain similar to that of the whole cohort. The nonlinear relationships were weaker, where modeling using fp or spline functions did not lead to a statistically significant improvement in model fit for both CST and total volume (Fig S8, available at www.ophthalmologyretina.org/). In addition, we also show the VA outcomes of ischemic CRVO defined in various ways in the whole LEAVO cohort and in this study sample in Table S9 (available at www.ophthalmologyretina.org/).

## Discussion

Our results show the determinants of final visual outcome at 52 and 100 weeks are similar. The predictors included age of patient at presentation of CRVO, baseline BCVA, and a gradable intact EZ. Participants aged < 75 years are more likely to gain BCVA and achieve > 70 letters at 52 weeks and 100 weeks. Although baseline CST and macular volume do not predict BCVA, our results show the relation of baseline CST and BCVA outcome for 10-letter gainers is not linear, and eyes with CST ≥ 750 μm and total macular volume > 14 mm^3^ have a decreasing probability of gaining ≥ 10 letters by 100 weeks. These observations persisted when the eyes with ischemic CRVO were excluded from the cohort, suggesting that these predictors also apply to eyes with ischemic CRVO at baseline that meet the LEAVO inclusion criteria and are continuously monitored every 4 to 8 weeks and treated on the basis of strict re-treatment criteria over 100 weeks. We deduced that this nonlinear effect may be due to the ceiling effect of baseline VA seen in participants with the lower extremity of CST, whereas participants with a moderately increased CST present with lower VA and have greater room for improvement and are more likely to gain 10 or more letters by 100 weeks. Those with extremely high baseline CST may have irreversible structural changes or coexistent ischemia and have a reduced odds of gaining 10 or more letters.

Previous CRVO studies found similar results regarding poor visual prognosis in older individuals.^17^^,^^18^ The SCORE study evaluated triamcinolone for ME due to CRVO at 12 months, and SCORE2, which treated central and hemiretinal vein occlusion with monthly aflibercept or bevacizumab, showed that younger patients are more likely to gain 15 letters at 6 months.^8^^,^^19^ The SHORE study analyzed predictors of VA gains after 7 monthly intravitreal ranibizumab injections, and reported younger age is a good predictor of visual gain.^20^ These observations suggest that irrespective of treatment type, regimen, or period of follow-up, older age is a poor visual prognostic indicator. The SCORE investigators hypothesized that photoreceptors in younger patients are more resilient to the acute insult of CRVO. Younger age is also associated with better visual prognosis in diabetic ME, further supporting the hypothesis that a young retina more readily withstands the acute and chronic insults of ME.^21^

We did not find duration of CRVO to be a favorable predictor of visual outcome probably because the majority (84%) of the study cohort were diagnosed with CRVO less than 3 months before randomization. The COPERNICUS and GALILEO studies showed that a CRVO diagnosis of 2 months or less had better visual outcomes compared with those diagnosed more than 2 months previously.^22^^,^^23^

Patients with lower baseline BCVA are more likely to gain 10 or more letters, but eyes with baseline BCVA < 55 letters are less likely to achieve > 70 letters at 52 or 100 weeks. Baseline BCVA is a known predictor of final visual outcome in several CRVO and diabetic ME studies. The SHORE study looked at time to achieve 20/40 or better or 15-letter gain within 3 months of treatment, whereas SCORE and SCORE2 evaluated predictors of 15-letter gainers at 12 and 6 months, respectively.^24^ Baseline BCVA is a predictor of final visual outcome independent of the drug used, treatment regimen followed, VA outcome measure, or study period.

Unlike studies on diabetic ME, quantitative OCT parameters at baseline do influence final BCVA, with a CST of > 900 μm less likely to be associated with gains ≥ 10 letters compared with CST ≤ 900 μm. This difference from diabetic ME is likely due to the more severe acute edema in CRVO at presentation. Although these eyes are also likely to present with poor vision, the finding that the potential for visual improvement of 10 or more letters in these eyes is limited suggests some element of irreversible damage. These eyes may indicate ischemic CRVO, although the RAVE study showed that visual prognosis can be improved with anti-VEGF in preproliferative ischemic CRVO.^25^

A definite intact EZ layer at baseline is a predictor of good final visual outcomes, and a definite loss of baseline EZ integrity is a poor prognostic indicator. However, if the EZ layer is ungradable or questionable at baseline, it carries no predictive value.

Strengths of this study include 100-week on protocol-based follow-up, and the predictors were adjusted for the type of anti-VEGF agents and the number of injections received. We also studied 10-letter gainers and demonstrated that a baseline CST of > 900 μm is a poor prognostic indicator.

### Study Limitations

A study limitation was that baseline angiographic macular nonperfusion status was not assessed. However, we found that disorganization of the inner retinal layers, a surrogate marker of nonperfusion, is not a predictor. A substantial proportion of eyes with CRVO-related ME are anti-VEGF dependent beyond 2 years, and it is unknown if these predictors can be applied beyond 2 years of treatment because the LEAVO study final visit was at 100 weeks. The RAVE study shows that most eyes with ischemic CRVO deteriorate as soon as anti-VEGF therapy is withdrawn,^25^ and so their course after stopping anti-VEGF therapy is different than that of eyes with nonischemic CRVO.

## Conclusions

At presentation of ME due to CRVO, older age is a predictor of poor visual outcome, and lower BCVA predicts 10-letter gainers and higher gains in BCVA, although it is a poor predictor of achieving > 70 BCVA letters. An intact subfoveal EZ predicts a BCVA letter score > 70 at 100 weeks, whereas CST > 900 μm is a poor prognostic indicator. This information is important to share with patients to determine appropriate and individualized anti-VEGF management plans up to 100 weeks.
