# Supplementary figures and images for: Predictors of Visual Acuity Outcomes after Anti–Vascular Endothelial Growth Factor Treatment for Macular Edema Secondary to Central Retinal Vein Occlusion
Source: Ophthalmol Retina. 2021 Nov;5(11):1115–24. doi: 10.1016/j.oret.2021.02.008 (PMC8565966; doi:10.1016/j.oret.2021.02.008)

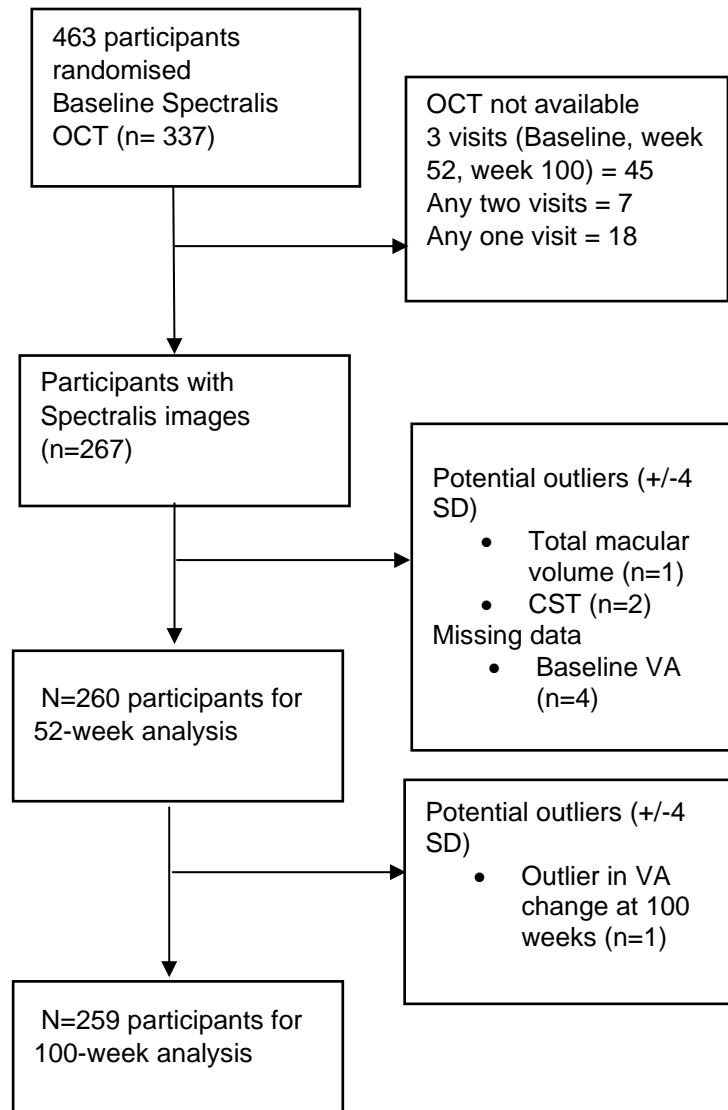

**eFigure 1: Participant flow chart**

Supplement: Fig S1 [file mmc1.pdf]

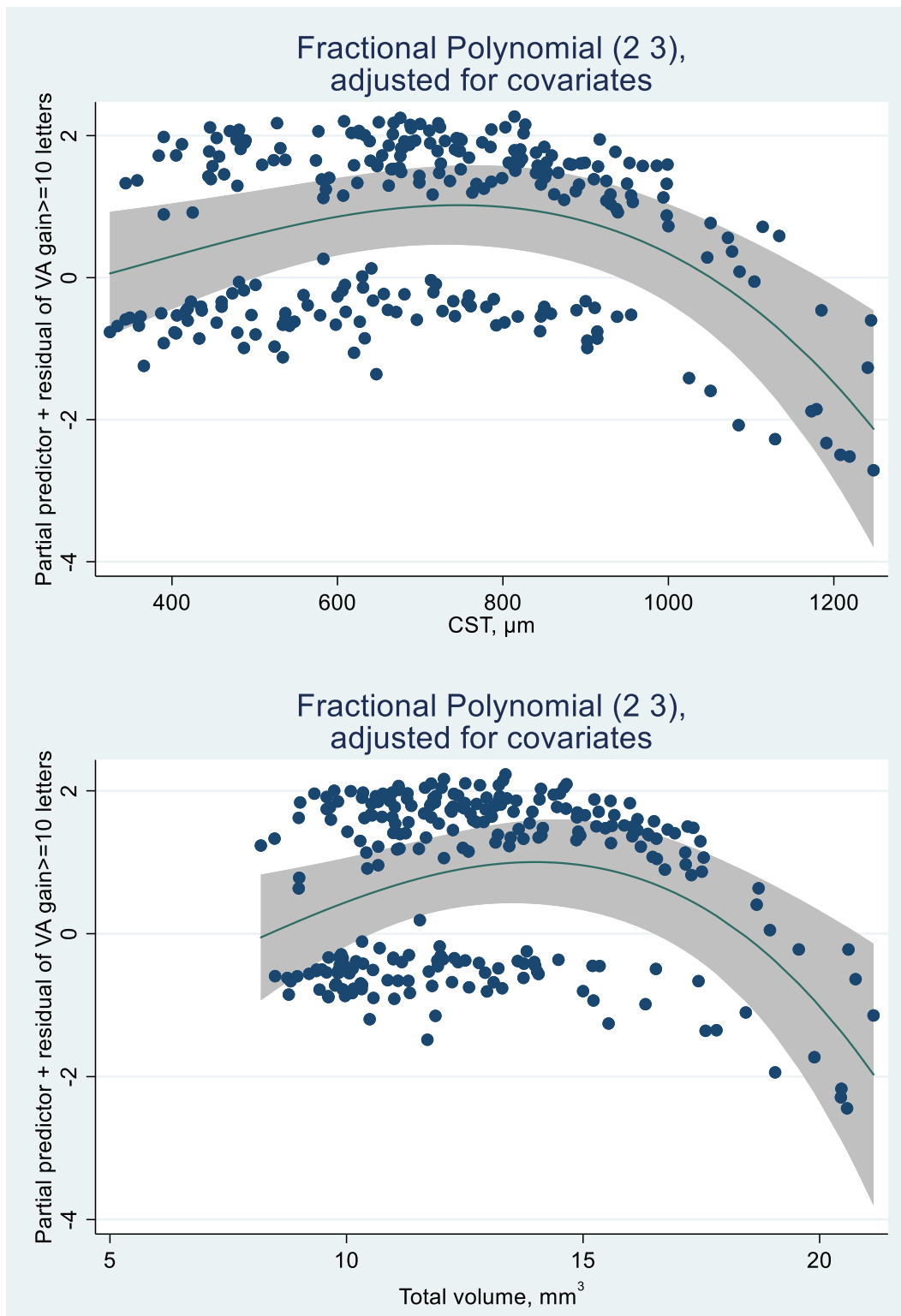

**eFigure 3: Fractional polynomial term for CST and Total volume at week 100 (multivariable analysis)**

Supplement: Fig S3 [file mmc3.pdf]
