# Supplementary material for: Predictors of Visual Acuity Outcomes after Anti–Vascular Endothelial Growth Factor Treatment for Macular Edema Secondary to Central Retinal Vein Occlusion
Source: Ophthalmol Retina. 2021 Nov;5(11):1115–24. doi: 10.1016/j.oret.2021.02.008 (PMC8565966; doi:10.1016/j.oret.2021.02.008)
Supplement: Fig S2 [file mmc2.pdf]

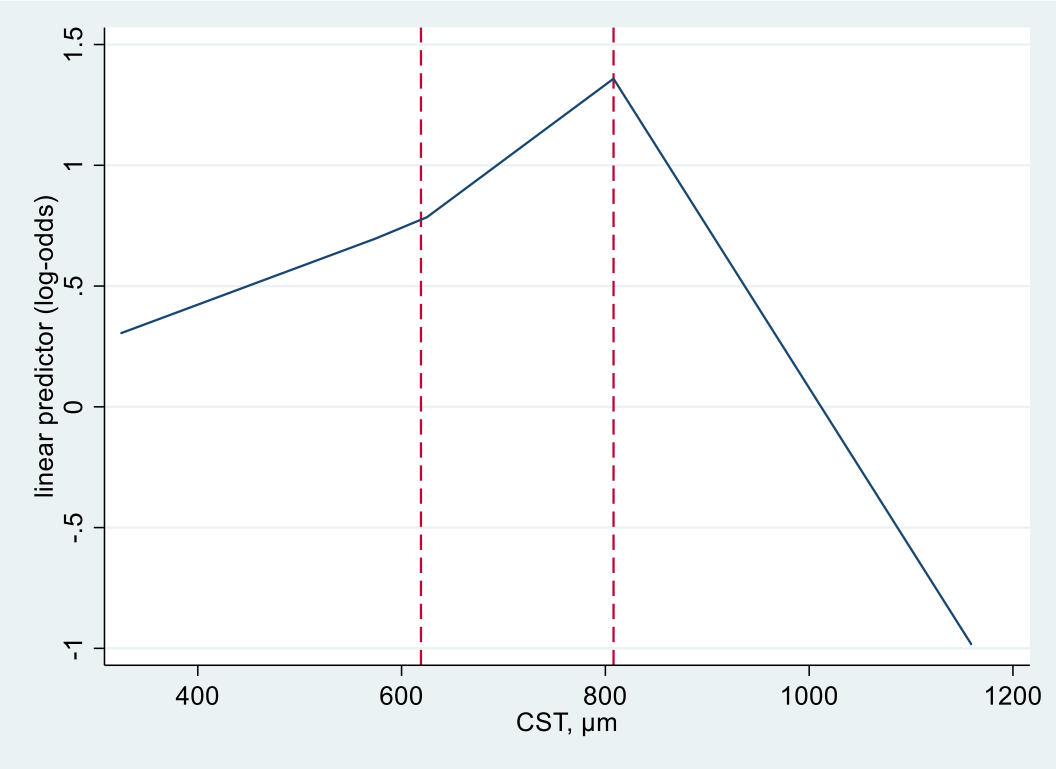

Splines were fitted in Stata, to model non-linearity in CST. Slopes corresponding to the piecewise linear functions were OR=1.002(0.997-1.006);p=0.502, OR=1.003(0.998-1.009);p=0.256 and OR=0.99 (0.989-0.997);p=0.001 with 2 knots placed at 619 and 808 microns (generated by the mkspline command in Stata version 16). Variance inflation factors were all <10 (1.73, 2.73 and 1.53 for the three piecewise polynomials). Model estimates for age, disease duration, baseline VA and treatment arm remain similar and consistent with analysis using fractional polynomials.

eFigure 2: Linear spline function for CST modelling 10-letter gainers at 100 weeks
