# Supplementary material for: Predictors of Visual Acuity Outcomes after Anti–Vascular Endothelial Growth Factor Treatment for Macular Edema Secondary to Central Retinal Vein Occlusion
Source: Ophthalmol Retina. 2021 Nov;5(11):1115–24. doi: 10.1016/j.oret.2021.02.008 (PMC8565966; doi:10.1016/j.oret.2021.02.008)
Supplement: Fig S4 [file mmc4.pdf]

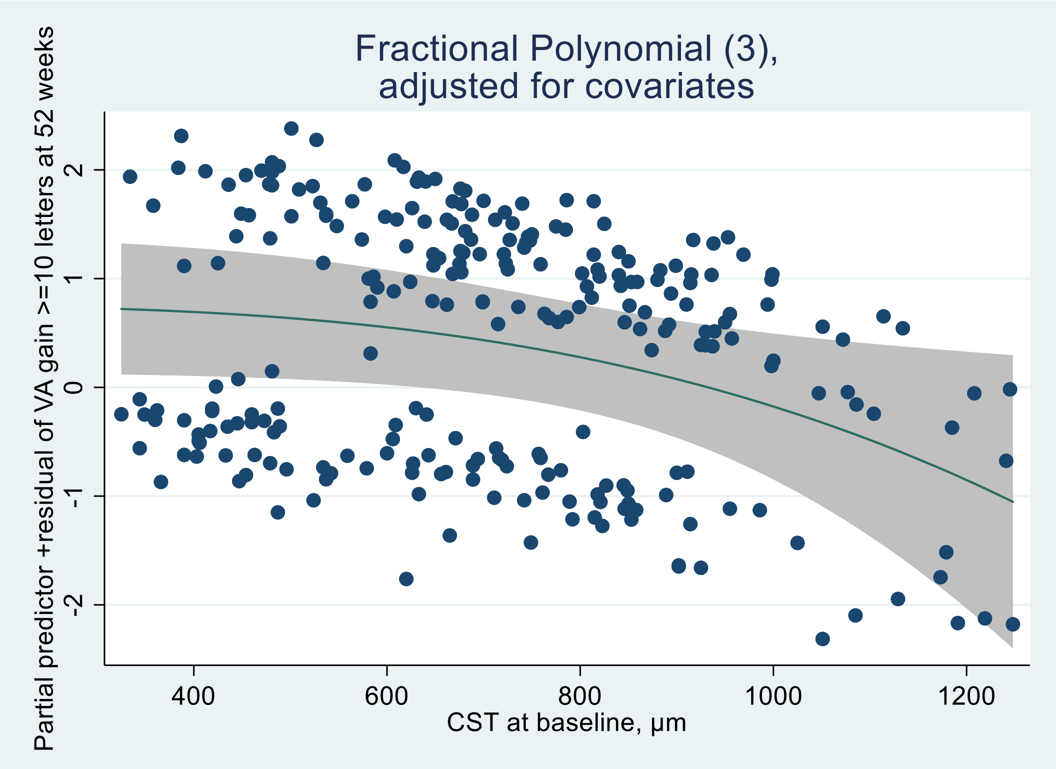

Abbreviations: VA, Visual Acuity; CST, central subfield thickness

eFigure 4: Fractional polynomial term for CST at week 52
