# Supplementary material for: Predictors of Visual Acuity Outcomes after Anti–Vascular Endothelial Growth Factor Treatment for Macular Edema Secondary to Central Retinal Vein Occlusion
Source: Ophthalmol Retina. 2021 Nov;5(11):1115–24. doi: 10.1016/j.oret.2021.02.008 (PMC8565966; doi:10.1016/j.oret.2021.02.008)
Supplement: Fig S5 [file mmc5.pdf]

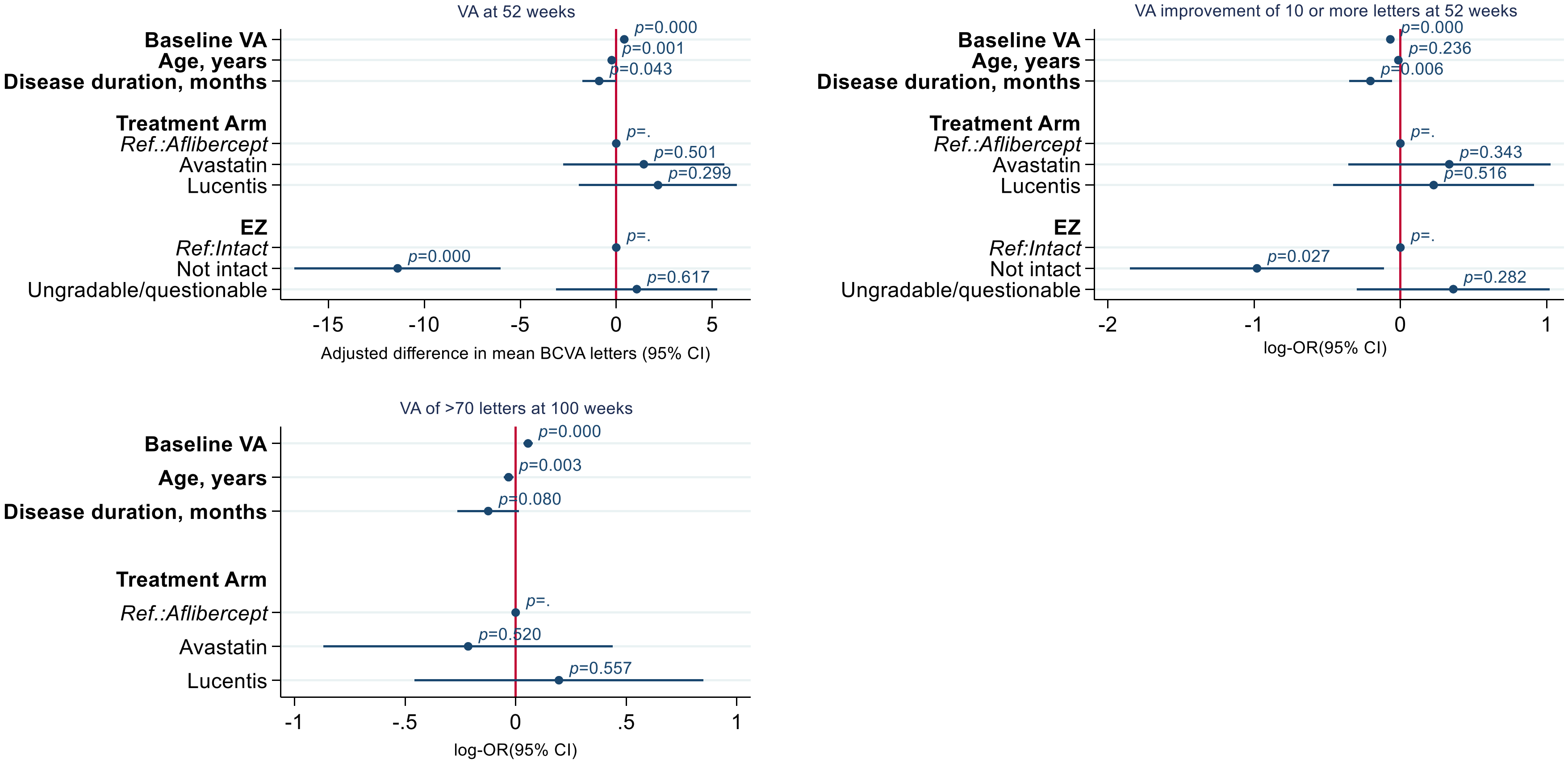

Abbreviations: VA, Visual Acuity; EZ, ellipsoid zone; CST, central subfield thickness. Variables that passed the p<0.1 threshold in the univariate (adjusted) analysis were subsequently included in the multivariable models (ELM, EZ and CST). Backward elimination was carried out locking in control variables or confounders for precision regardless of statistical significance and setting the variable elimination threshold at p<0.05. In all models ELM was eliminated at the 5% level (not presented), and in the model predicting VA gain of 10 or more letters CST was eliminated.

eFigure 5: Results from multivariable analysis for week 52 analysis
