# Supplementary material for: Predictors of Visual Acuity Outcomes after Anti–Vascular Endothelial Growth Factor Treatment for Macular Edema Secondary to Central Retinal Vein Occlusion
Source: Ophthalmol Retina. 2021 Nov;5(11):1115–24. doi: 10.1016/j.oret.2021.02.008 (PMC8565966; doi:10.1016/j.oret.2021.02.008)
Supplement: Fig S6 [file mmc6.pdf]

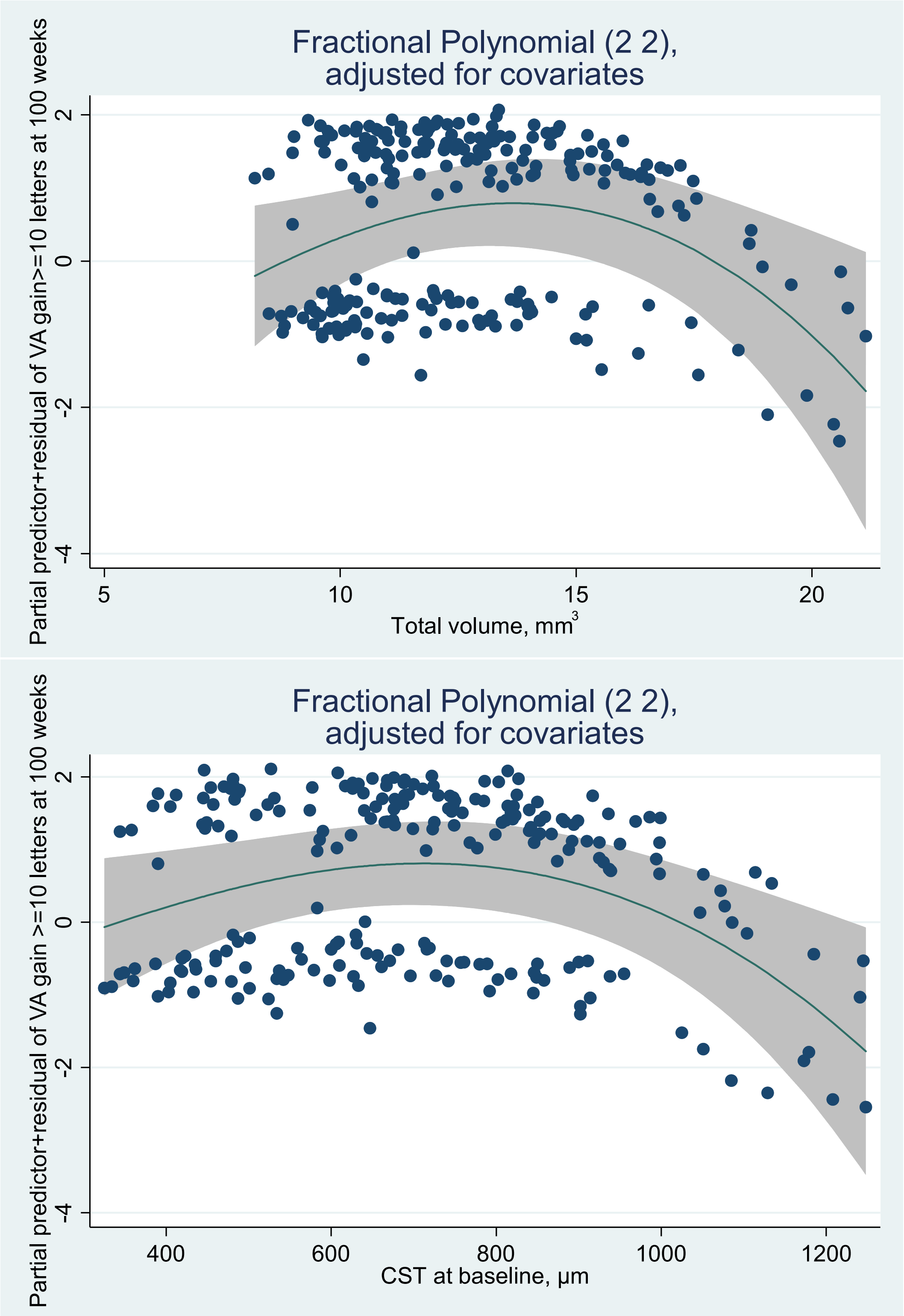

eFigure 6. Fractional polynomial terms selected excluding participants with ischemic CRVO at baseline for outcome of 10-letter gainers at 100 weeks, with an optimal fp selection criterion at alpha=0.1
