# Supplementary material for: Predictors of Visual Acuity Outcomes after Anti–Vascular Endothelial Growth Factor Treatment for Macular Edema Secondary to Central Retinal Vein Occlusion
Source: Ophthalmol Retina. 2021 Nov;5(11):1115–24. doi: 10.1016/j.oret.2021.02.008 (PMC8565966; doi:10.1016/j.oret.2021.02.008)
Supplement: Fig S7 [file mmc7.pdf]

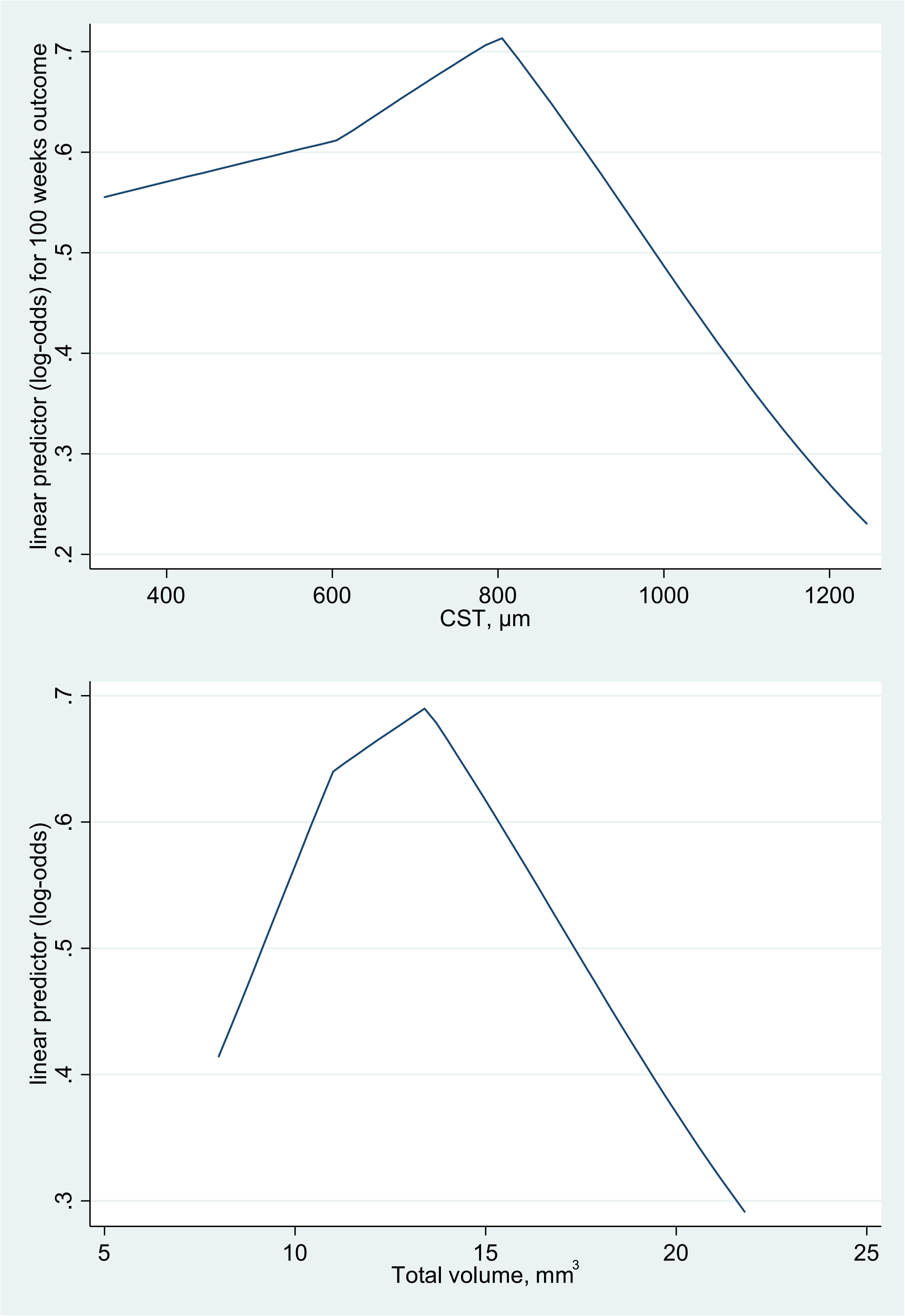

eFigure 7. Linear splines for modelling CT and total volume, for the outcome of gaining by 10 or more ETDRS letters by week 100, after excluding participants with ischemic CRVO at baseline, with 3 knots equally spaced across the percentiles of the data

For CST; slopes(beta coefficients) corresponding to piecewise linear functions were OR=1.00(1.00-1.01);p=0.698, OR=1.003(1.00-1.01);p=0.316 and 0.99(0.99-1.00);p=0.005 with Knots placed at 608, 803 µm. For total volume; slopes (beta coefficients) corresponding to piecewise linear functions were OR=1.44(0.78-2.64);p=0.243, OR=1.11(0.74-1.67);p=0.611 and OR=0.79(0.62-1.00);p=0.053 with Knots placed at 11.01, 13.44 mm^3.
