# Supplementary material for: Predictors of Visual Acuity Outcomes after Anti–Vascular Endothelial Growth Factor Treatment for Macular Edema Secondary to Central Retinal Vein Occlusion
Source: Ophthalmol Retina. 2021 Nov;5(11):1115–24. doi: 10.1016/j.oret.2021.02.008 (PMC8565966; doi:10.1016/j.oret.2021.02.008)
Supplement: Fig S8 [file mmc8.pdf]

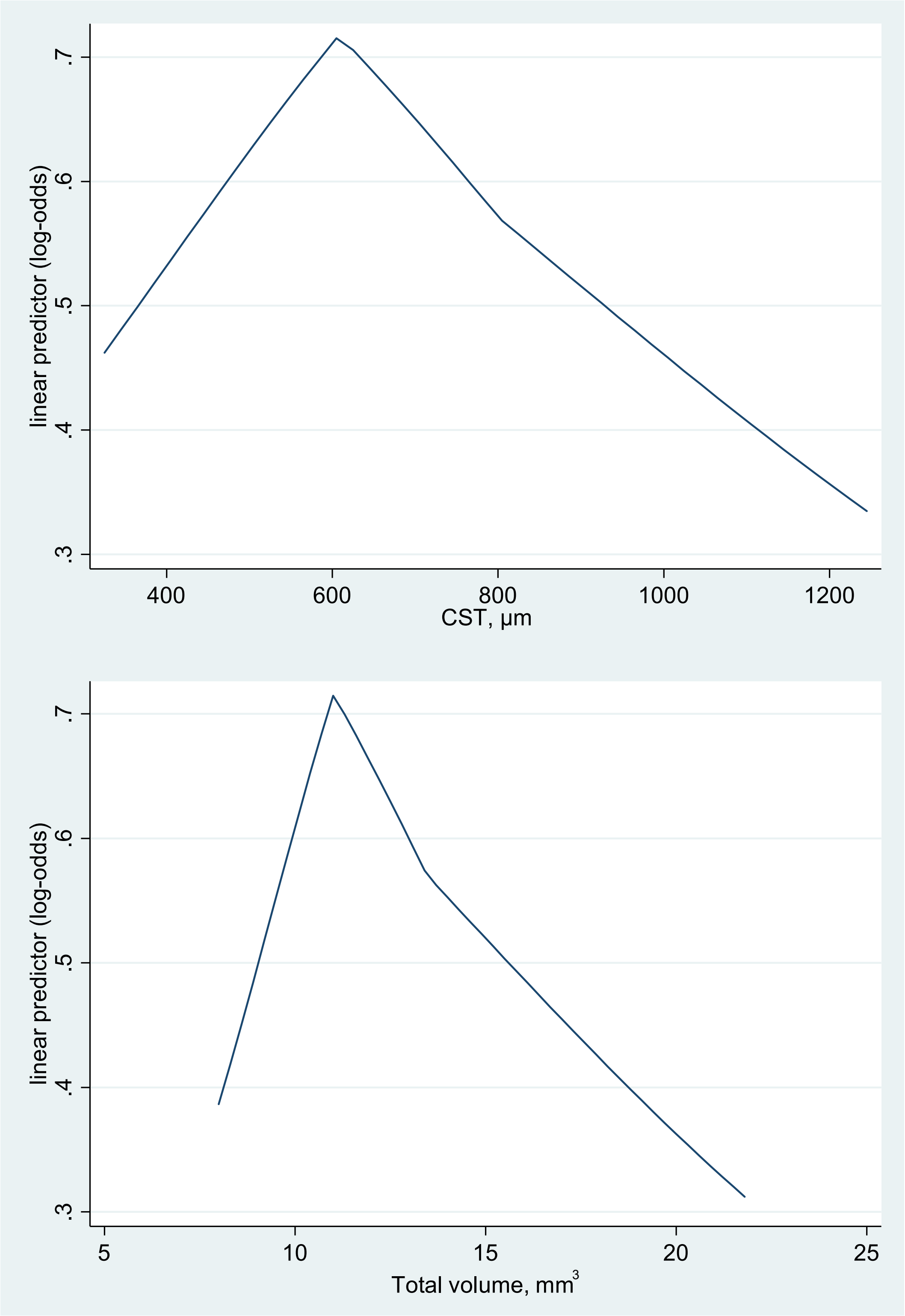

eFigure 8. Linear splines for modelling CT and total volume, for the outcome of gaining by 10 or more ETDRS letters by week 52, after excluding participants with ischemic CRVO at baseline, with 3 knots equally spaced across the percentiles of the data

For CST; slopes(beta coefficients) corresponding to piecewise linear functions were OR=1.005(1.00-1.01);p=0.075, OR=1.00(0.99-1.00);p=0.147 and 1.00(0.99-1.00);p=0.174 with Knots placed at 608, 803 µm. For total volume; slopes (beta coefficients) corresponding to piecewise linear functions were OR=1.78(0.95-3.35);p=0.072, OR=0.73(0.48-1.10);p=0.132 and OR=0.85(0.66-1.09);p=0.194 with Knots placed at 11.01, 13.47 mm^3.
