# Supplementary material for: Predictors of Visual Acuity Outcomes after Anti–Vascular Endothelial Growth Factor Treatment for Macular Edema Secondary to Central Retinal Vein Occlusion
Source: Ophthalmol Retina. 2021 Nov;5(11):1115–24. doi: 10.1016/j.oret.2021.02.008 (PMC8565966; doi:10.1016/j.oret.2021.02.008)
Supplement: Table S1 [file mmc9.pdf]

Supplementary file

eTable 1: Definitions of OCT parameters

OCT parameters	Grading definition
Epiretinal membrane (ERM)[1]	The ERM was deemed present if a macular pseudo-hole, a difference in optical reflectivity between membrane and retina or a visible membrane tuft or edge was observed.
Vitreomacular traction (VMT)[2]	VMT was defined anomalous posterior vitreous detachment identified as highly reflective band on the surface of the retina attached at specific sites and elevated off the surface elsewhere, associated with anatomic distortion of fovea
Disorganization of the inner retinal layers (DRIL)[3]	An area of the inner retina where the boundary between the ganglion cell layer inner plexiform layer complex, inner nuclear layer and outer plexiform layer could not be identified in the central 5-line scans. If the average across 5-line scan ≥50%, DRIL was considered positive.
Macular oedema[4]	Diffuse retinal thickening defined as sponge-like retinal swelling with reduced intra-retinal reflectivity and the absence of hypo reflective spaces; cystoid macula edema (CME) defined as intra-retinal cystoid spaces of low reflectivity with highly reflective septa separating cystoid-like cavities and mixed oedema when cystoid and diffuse edema co-existed. Intra-retinal cysts in CME and mixed edema were considered as small, <250mm, medium ≥250mm and <500mm, and large ≥500mm based on the greatest horizontal diameter
Hyper reflective foci (HRF)[5]	Small (<30µm), hyper reflective intraretinal spots distributed throughout any or all retinal layers without showing a characteristic intra-retinal location. Presence of 30 or more HRF was quantified as 'present'.
External limiting membrane (ELM)[6]	The faint narrow line superior to the ellipsoid zone and was graded as intact if visible throughout the entire foveal line scan, not intact if disrupted or completely absent under high contrast settings, and ungradable due to shadowing of edematous retina. If graders could visualize the ELM but could not define its integrity, it was recorded as questionable
Ellipsoid zone (EZ)[7]	The third hyper reflective band and is a distinct band just above the high-reflectance layer of the retinal pigment epithelium–choriocapillaris complex were graded similar to the ELM
Sub retinal detachment (SRD)[8]	Shallow elevation of the retina, with an optically clear space between the retina and the retinal pigment epithelium. SRD was defined as cannot grade, definite, questionable or no evidence.

References

1. Wilkins JR, Puliafito CA, Hee MR, et al. Characterization of epiretinal membranes using optical coherence tomography. *Ophthalmology.* 1996;103(12):2142-2151.
2. Duker JS, Kaiser PK, Binder S, et al. The International Vitreomacular Traction Study Group classification of vitreomacular adhesion, traction, and macular hole. *Ophthalmology.* 2013;120(12):2611-2619.
3. Sun JK, Radwan SH, Soliman AZ, et al. Neural Retinal Disorganization as a Robust Marker of Visual Acuity in Current and Resolved Diabetic Macular Edema. *Diabetes.* 2015;64(7):2560-2570.
4. Sun JK, Lin MM, Lammer J, et al. Disorganization of the retinal inner layers as a predictor of visual acuity in eyes with center-involved diabetic macular edema. *JAMA Ophthalmol.* 2014;132(11):1309-1316.
5. Vujosevic S, Bini S, Torresin T, et al. Hyperreflective retinal spots in normal and diabetic eyes: B-Scan and En Face Spectral Domain Optical Coherence Tomography Evaluation. *Retina.* 2017;37(6):1092-1103.
6. Landa G, Gentile RC, Garcia PM, Muldoon TO, Rosen RB. External limiting membrane and visual outcome in macular hole repair: spectral domain OCT analysis. *Eye (Lond).* 2012;26(1):61-69.
7. Panozzo G, Cicinelli MV, Augustin AJ, et al. An optical coherence tomography-based grading of diabetic maculopathy proposed by an international expert panel: The European School for Advanced Studies in Ophthalmology classification. *Eur J Ophthalmol.* 2020;30(1):8-18.
8. Hu Y, Wu Q, Liu B, et al. Comparison of clinical outcomes of different components of diabetic macular edema on optical coherence tomography. *Graefes Arch Clin Exp Ophthalmol.* 2019;257(12):2613-2621.
