# Supplementary material for: Predictors of Visual Acuity Outcomes after Anti–Vascular Endothelial Growth Factor Treatment for Macular Edema Secondary to Central Retinal Vein Occlusion
Source: Ophthalmol Retina. 2021 Nov;5(11):1115–24. doi: 10.1016/j.oret.2021.02.008 (PMC8565966; doi:10.1016/j.oret.2021.02.008)
Supplement: Table S2 [file mmc10.pdf]

eTable 2. Correlation table, showing p-values of intercorrelations for all baseline risk factors

	Age	Gender	Disease duration	BCVA	CST	Total Volume	SRD	DRIL	EZ
**Gender**	0.91								
**Disease duration**	0.17	0.67							
**BCVA**	0.57	0.42	0.18						
**CST**	*0.005*[a]	0.77	0.30	*<0.001*[a]					
**Total Volume**	*0.002*[a]	0.82	0.83	*<0.001*[a]	*<0.001*				
**SRD**	0.29	0.38	0.55	0.33	*<0.001*	*0.001*			
**DRIL**	0.05	0.73	0.78	*<0.001*	*<0.001*	*<0.001*	0.37		
**EZ**	*<0.001*	0.81	0.47	*<0.001*	*<0.001*	*<0.001*	*0.004*	*<0.001*	
**ELM**	*0.002*	0.76	0.11	*<0.001*	*<0.001*	*<0.001*	0.16	*<0.001*	*<0.001*

Abbreviations: OCT, optical coherence tomography; BCVA, Best Corrected Visual Acuity; CST, Central subfield thickness; SRD, subretinal detachment; DRIL, disorganisation of retinal inner layers; EZ, ellipsoid zone; ELM, external limiting membrane;
Continuous vs continuous associations assessed by spearman's correlation test
Continuous vs categorical associations assessed by Kruskal-Wallis test
Categorical vs categorical associations assessed by χ^2 test; fishers test used on those with >20% cells with expected count <5
Statistically significant p-values($p<0.05$) have been *italicized*
[a] Negative (inverse) correlation (for Age vs; CST, spearman's correlation coefficient ρ=-0.21; Total volume, ρ= 0.20. For BCVA vs; CST, ρ = -0.52, Total volume, ρ= -0.53)
