# Supplementary material for: Predictors of Visual Acuity Outcomes after Anti–Vascular Endothelial Growth Factor Treatment for Macular Edema Secondary to Central Retinal Vein Occlusion
Source: Ophthalmol Retina. 2021 Nov;5(11):1115–24. doi: 10.1016/j.oret.2021.02.008 (PMC8565966; doi:10.1016/j.oret.2021.02.008)
Supplement: Table S3 [file mmc11.pdf]

eTable 3: Dunn's test of multiple comparisons using rank sums between CST and EZ following Kruskal-Wallis test of stochastic dominance among 4 groups

	EZ		ELM	
**EZ/ELM**	**1- intact**	**2- Not Intact**	**1- Intact**	**2- Not Intact**
**2- not Intact**	*<0.001*		*<0.001*	
**3- Ungradable/questionable**	*<0.001*	0.2505	*<0.001*	0.1528

Type-1 error due to multiple comparisons was handled by adjustment of the p-values have via the Bonferroni method
Abbreviations: EZ, ellipsoid zone; ELM, external limiting membrane
