# Supplementary material for: Predictors of Visual Acuity Outcomes after Anti–Vascular Endothelial Growth Factor Treatment for Macular Edema Secondary to Central Retinal Vein Occlusion
Source: Ophthalmol Retina. 2021 Nov;5(11):1115–24. doi: 10.1016/j.oret.2021.02.008 (PMC8565966; doi:10.1016/j.oret.2021.02.008)
Supplement: Table S4 [file mmc12.pdf]

eTable 4: Vision outcomes at 52 weeks, by baseline demographic characteristics and VA

Clinical / OCT characteristics at baseline	Mean change in BCVA from baseline to 100 weeks or final BCVA at week 100[a]		BCVA improvement ≥10 letters		Final BCVA >70 letters	
	**Estimate (95% CI)**	**p-value**	**OR (95% CI)**	**p-value**	**OR (95% CI)**	**p-value**
Age						
<50	Ref	-	Ref	-	Ref	-
50-74	-2.69(-9.42,4.05)	0.43	0.87(0.29, 2.60)	0.80	0.57(0.20, 1.62)	0.29
≥75	-10.61(-17.56, -3.66)	*0.003*	0.52(0.17, 1.58)	0.25	0.29(0.10, 0.86)	0.03
Age (linear)	-0.29(-0.43, -0.16)	*<0.001*	0.98(0.96, 1.00)	*0.04*	0.97(0.95, 0.99)	0.002
Disease duration	-1.16(-2.08, -0.24)	*0.01*	0.80(0.69, 0.92)	*0.003*	0.87(0.76, 1.00)	0.05
Sex						
Males	Ref	-	Ref	-	Ref	-
Females	-1.61(-5.33,2.11)	0.39	0.80(0.47, 1.37)	0.42	0.64(0.38, 1.08)	0.10
BCVA						
>70 letters	Ref	-	Ref	-	-	-
55-70 letters	-7.45(-13.67, -1.23)	0.02	2.40(1.00,5.75)	0.05	Ref	-
37- 54 letters	-15.56(-22.29, -8.84)	*<0.001*	6.41(2.56,16.01)[b]	*<0.001*	0.33(0.18,0.62)[c]	*<0.001*
<37 letters	-21.00(-28.33, -13.66)	*<0.001*	-		0.19(0.09,0.42)	*<0.001*
BCVA (linear)	0.42(0.29,0.55)	*<0.001*	0.94(0.92,0.96)	*<0.001*	1.05(1.03,1.08)	*<0.001*

All p-values adjusted for baseline VA and treatment arm

[a] For interpreting baseline VA, the outcome should be interpreted as the final visual acuity at 100 weeks

[b] Groups 37-54 and <37 were collapsed for outcome BCVA improvement >=10 letters due to reduced sample size in group <37 letters that did not improve

[c] Groups >70 and 55-70 were collapsed for outcome final VA>70 due to reduced sample size in group >70
