# Supplementary material for: Predictors of Visual Acuity Outcomes after Anti–Vascular Endothelial Growth Factor Treatment for Macular Edema Secondary to Central Retinal Vein Occlusion
Source: Ophthalmol Retina. 2021 Nov;5(11):1115–24. doi: 10.1016/j.oret.2021.02.008 (PMC8565966; doi:10.1016/j.oret.2021.02.008)
Supplement: Table S5 [file mmc13.pdf]

eTable 5: Vision outcomes at 52 weeks, by baseline ocular features (OCT variables)

**Clinical / OCT characteristic at baseline**	**Mean change in BCVA from baseline to 100 weeks**		**BCVA improvement ≥10 letters**		**Final BCVA of >70 letters**	
	**Mean difference (95% CI)**	**p-value**	**OR (95% CI)**	**p-value**	**OR (95% CI)**	**p-value**
**CST (linear/FP terms)**	-0.01(-0.02,0.001)	0.08	FP Term 1[a]	*0.03*	1.00(0.998,1.001)	0.23
**SRD**						
Absence	Ref	-	Ref	-	Ref	-
Presence	-3.15(-7.02,0.71)	0.11	1.13(0.61,2.08)	0.70	0.59(0.32,1.07)	0.08
**EZ**						
Intact	Ref	-	Ref	-	Ref	-
Not Intact	-11.39(-16.76, -6.02)	*<0.001*	0.38(0.16,0.89)	*0.03*	0.45(0.19,1.07)	0.07
Ungradable/questionable	1.07(-3.13,5.27)	0.62	1.44(0.74,2.78)	0.28	1.34(0.69,2.60)	0.39
**ELM**						
Intact	Ref	-	Ref	-	Ref	-
Not Intact	-6.45(-12.95, 0.059)	0.05	0.51(0.18,1.40)	0.19	1.04(0.38,2.85)	0.94
Ungradable/questionable	2.75(-1.22,6.73)	0.17	1.61(0.88,2.97)	0.13	1.46(0.80,2.69)	0.39

Only those variables with statistically significant p-values at the 10% threshold (p<0.1) have been presented. Statistically significant p-values at 5% threshold (p<0.05) have been italicized
Ungradable and inconclusive observations were combined into a single category, where numbers were <5% results were not presented or dropped entirely to enable stable model estimation
Abbreviations: FP, fractional polynomial; OCT; BCVA, best corrected visual Acuity; CST, Central subfield thickness; SRD, Subretinal detachment ;EZ, ellipsoid zone ;ELM, external limiting membrane ; ERM, epiretinal membrane
Fractional polynomial terms:
[a] CST, Term 1; X^3-361.22
