# Supplementary material for: Predictors of Visual Acuity Outcomes after Anti–Vascular Endothelial Growth Factor Treatment for Macular Edema Secondary to Central Retinal Vein Occlusion
Source: Ophthalmol Retina. 2021 Nov;5(11):1115–24. doi: 10.1016/j.oret.2021.02.008 (PMC8565966; doi:10.1016/j.oret.2021.02.008)
Supplement: Table S6 [file mmc14.pdf]

eTable 6. Table comparing visual acuity outcomes in participants with ischemic vs non-ischemic CRVO at baseline

Characteristic		Non-ischemic (n=239)		Ischemic (n=28)	
		N	Median(IQR)/ Mean(SD) /N(%)	N	Median(IQR)/Mean(SD) / N(%)
**Baseline VA**<br>Median(IQR)<br>Mean(SD)		236	 58 (50-66.5)<br>55.7(13.8)	27	 37(28-52)<br>41.3(15.6)
**CST, µm**<br>Median(IQR)<br>Mean(SD)		236	 695(537-852)<br>714.9(221.4)	28	 761(634-941.5)<br>792.3(263.9)
**Total volume, mm^3**<br>Median(IQR)<br>Mean(SD)		236	 12.3(10.6-14.4)<br>12.8(2.9)	26	 14.1(11.5-16.5)<br>14.2(3.0)
**100-week visual acuity outcomes**	**100-week VA**<br>Median(IQR)<br>Mean(SD)	239	 71 (58-80)<br>67.0(17.3)	28	 68.5 (61.5-79)<br>65.7(17.5)
	**Change in VA**<br>Median(IQR)<br>Mean(SD)	236	 12.5(3-22)<br>11.5(19.1)	27	 24(12-39)<br>24.3(19.7)
	**% 10-letter gainers**	236	142(60.2%)	27	21(77.8%)
	**% achieving >70 letters**	239	123(51.5%)	28	12(42.9%)
**52-week visual acuity outcomes**	**52-week VA**<br>Median(IQR)<br>Mean(SD)	239	 70(59-79)<br>67.4(15.8)	28	 71.5(55.5-77.5)<br>65.3(18.4)
	**Change in VA**<br>Median(IQR)<br>Mean(SD)	236	 12.5(4-20)<br>11.9(16.8)	27	 25(9-39)<br>24.0(18.4)
	**% 10-letter gainers**	236	138(58.5%)	27	20(74.1%)
	**% achieving >70 letters**	239	119(49.8%)	28	16(57.1%)

Abbreviations: VA, Visual acuity; CST, central-subfield thickness
