# Supplementary material for: Predictors of Visual Acuity Outcomes after Anti–Vascular Endothelial Growth Factor Treatment for Macular Edema Secondary to Central Retinal Vein Occlusion
Source: Ophthalmol Retina. 2021 Nov;5(11):1115–24. doi: 10.1016/j.oret.2021.02.008 (PMC8565966; doi:10.1016/j.oret.2021.02.008)
Supplement: Table S7 [file mmc15.pdf]

eTable 7. Visual acuity outcomes at 100 weeks, by demographic variables, baseline BCVA and OCT characteristics after excluding participants with ischemic CRVO at baseline

**Patient characteristic**	**Final BCVA at week 100[a]**		**BCVA improvement ≥10 letters**		**Final BCVA >70 letters**	
	**Estimate (95% CI)**	**p-value**	**OR (95% CI)**	**p-value**	**OR (95% CI)**	**p-value**
			**Demography and baseline VA**			
**Age [b]**<br><50<br>50-74<br>≥75<br><br>**Age [b]**<br>(linear)	 Ref<br>-5.92(-13.73,1.90)<br>-12.77(-20.80,-4.74)<br><br><br>-0.32(-0.47,-0.16)	 -<br>0.14<br>*0.002*<br><br><br>*<0.001*	 Ref<br>0.66(0.19,2.27)<br>0.34(0.10,1.18)<br><br><br>0.97(0.94,0.99)	 -<br>0.51<br>0.09<br><br><br>*0.005*	 Ref<br>0.22(0.06,0.77)<br>0.15(0.04,0.53)<br><br><br>0.97(0.95,0.99)	 -<br>*0.02*<br>*0.003*<br><br><br>*0.003*
**Disease duration [b]**	-0.87(-1.96,0.22)	0.12	0.88(0.76,1.02)	0.09	0.93(0.81,1.07)	0.29
**Sex [b]**<br>Males<br>Females	 Ref<br>-1.56(-5.74,2.62)	 -<br>0.46	 Ref<br>0.95(0.55,1.66)	 -<br>0.86	 Ref<br>1.17(0.68,2.00)	 -<br>0.57
**BCVA, letters [b]**<br>>70<br>55-70<br>37- 54<br><37<br><br>**BCVA [b]**<br>(linear)	 <br>Ref<br>-7.10(-13.83,-0.36)<br>-13.90(-21.31,-6.48)<br>-16.66(-25.27,-8.05)<br><br>0.36(0.21,0.51)	 <br>-<br>*0.04*<br>*<0.001*<br>*<0.001*<br><br>*<0.001*	 <br>Ref<br>2.10(0.90,4.91)<br>4.24(1.71,10.49)[c]<br>-<br><br>0.95(0.93,0.97)	 <br>-<br>0.09<br>*0.002*<br><br><br>*<0.001*	 <br>Ref<br>0.63(0.25,1.55)<br>0.22(0.08,0.59)<br>0.16(0.05,0.52)<br><br>1.04(1.02,1.07)	 <br>-<br>0.31<br>*0.003*<br>*0.002*<br><br>*<0.001*
			**OCT characteristics [d]**			
**CST, µm [e]**<br><br>Linear/FP terms	-0.008(-0.02,0.004)	0.17	2 FP terms [f]	*0.012*<br>*0.009*	1.00(1.00,1.002)	0.88
**Volume, mm³ [e]**	-0.27(-1.16,0.62)	0.55	2 FP terms [g]	*0.018*<br>*0.010*	1.03(0.91,1.16)	0.69
**SRD[e]**<br>Absence<br>Presence	 Ref<br>-1.85(-6.23,2.53)	 -<br>0.41	 Ref<br>0.76(0.40,1.44)	 -<br>0.40	 Ref<br>0.98(0.54,1.80)	 -<br>0.96
**DRIL[e]**<br>Absent<br>Present	 Ref<br>-0.60(-5.08,3.88)	 -<br>0.79	 Ref<br>0.92(0.49,1.75)	 -<br>0.81	 Ref<br>1.22(0.65,2.28)	 -<br>0.53
**EZ [e]**<br>Intact<br>Not Intact<br>Ungradable/Questionable	 Ref<br>-15.41(-21.31,-9.43)<br>0.47(-4.20,5.13)	 -<br>*<0.001*<br>0.84	 Ref<br>0.19(0.07,0.52)<br>1.74(0.87,3.51)	 -<br>*0.001*<br>0.12	 Ref<br>0.21(0.07,0.60)<br>2.11(1.04,4.30)	 -<br>*0.003*<br>*0.04*
**ELM [e]**<br>Intact<br>Not Intact<br>Ungradable/Questionable	 Ref<br>-9.20(-16.68,-1.72)<br>2.43(-2.08,6.94)	 -<br>*0.02*<br>0.29	 Ref<br>0.39(0.13,1.15)<br>1.55(0.82,2.95)	 -<br>0.09<br>0.18	 Ref<br>0.51(0.17,1.55)<br>2.17(1.14,4.15)	 -<br>0.24<br>*0.02*

1 bivariate outlier was identified in VA change (67 letters drop) after truncating at 3SD and 3 further outliers identified and removed from CST and total volume

28 participants with ischemic CRVO at baseline were excluded

[a] For baseline VA, the outcome should be interpreted as the final visual acuity at 100 weeks

[b] Adjusted for baseline VA and treatment arm
[c] Groups 37-54 and <37 was collapsed for outcome due to low numbers in group <37 letters that did not improve.
[d] Showing only variables that were statistically significant at the 10% threshold ($p<0.1$).
[e] Adjusted for baseline VA, age, disease duration and treatment arm
Statistically significant p-values at the 5% threshold ($p<0.05$) have been italicized.
Fractional polynomial terms:
[f] CST: Term 1 = X^2-49.91, Term 2= X^2*ln(X)-97.57 (where: X = CST/100); fp model AIC=283 vs linear model AIC=289
[g] Total volume: Term 1= X^2-1.62 , Term 2= X^2*ln(X)-0.39 (where: X = total volume/10); fp model AIC=284 vs linear model AIC=290
LR-test comparing linear and non-linear models (p=0.005; LR χ^2=8.00 for CST and p=0.009; LR χ^2=6.92 for total volume from a likelihood ratio test). Model AIC is interpreted as an out-of-sample prediction error and can used to compare nested models
Abbreviations: FP, fractional polynomial; OCT; BCVA, best corrected visual Acuity; CST, Central subfield thickness; SRD, sub-retinal detachment; DRIL, disorganization of retinal inner layers; EZ, ellipsoid zone; ELM, external limiting membrane; AIC, Akaike information criterion; LR, Likelihood-ratio test
