# Supplementary material for: Predictors of Visual Acuity Outcomes after Anti–Vascular Endothelial Growth Factor Treatment for Macular Edema Secondary to Central Retinal Vein Occlusion
Source: Ophthalmol Retina. 2021 Nov;5(11):1115–24. doi: 10.1016/j.oret.2021.02.008 (PMC8565966; doi:10.1016/j.oret.2021.02.008)
Supplement: Table S8 [file mmc16.pdf]

**eTable 8.** **Visual acuity outcomes at 52 weeks, by demographic variables, baseline BCVA and OCT characteristics after excluding participants with ischemic CRVO at baseline**

| **Patient characteristic** | **Final BCVA at week 100[a]** | | **BCVA improvement ≥10 letters** | | **Final BCVA >70 letters** | |
|---|---|---|---|---|---|---|
| | **Estimate (95% CI)** | **p-value** | **OR (95% CI)** | **p-value** | **OR (95% CI)** | **p-value** |
| **Demography and baseline VA** | | | | | | |
| **Age [b]** | | | | | | |
| <50 | Ref | - | Ref | - | Ref | - |
| 50-74 | -4.33(-11.41,2.75) | 0.23 | 0.80(0.24,2.62) | 0.71 | 0.55(0.17,1.71) | 0.30 |
| ≥75 | -12.27(-19.55,-4.99) | *0.001* | 0.48(0.14,1.62) | 0.24 | 0.26(0.08,0.85) | *0.03* |
| **Age [b]** (linear) | -0.31(-0.45,-0.17) | *<0.001* | 0.98(0.95,1.00) | 0.05 | 0.97(0.94,0.99) | *0.004* |
| **Disease duration [b]** | -0.77(-1.77,0.23) | 0.13 | 0.81(0.70,0.95) | *0.01* | 0.90(0.78,1.04) | 0.16 |
| **Sex [b]** | | | | | | |
| Males | Ref | - | Ref | - | Ref | - |
| Females | -1.49(-5.32,2.33) | 0.44 | 0.74(0.42,1.29) | 0.29 | 0.71(0.41,1.23) | 0.22 |
| **BCVA, letters [b]** | | | | | | |
| >70 | Ref | - | Ref | - | - | - |
| 55-70 | -7.63(-13.75,-1.51) | *0.02* | 2.49(1.03,6.00) | *0.04* | Ref [c] | - |
| 37- 54 | -16.82(-23.56,-10.08) | *<0.001* | 5.94(2.32,15.21)[c] | *<0.001* | 0.30(0.15,0.57) | *<0.001* |
| <37 | -20.31(-28.13,-12.48) | *<0.001* | - | | 0.16(0.06,0.43) | *<0.001* |
| **BCVA [b]** (linear) | 0.43(0.29,0.57) | *<0.001* | 0.94(0.91,0.96) | *<0.001* | 1.06(1.04,1.09) | *<0.001* |
| **OCT characteristics [d]** | | | | | | |
| **CST, µm [e]** linear | -0.01(-0.02,0.003) | 0.14 | 1.00(1.00,1.00) | 0.24 | 1.00(1.00-1.00) | 0.50 |
| **Volume, mm$^3$ [e]** | -0.58(-1.39,0.23) | 0.16 | 0.90(0.78,1.03) | 0.13 | 0.95(0.83,1.08) | 0.40 |
| **SRD[e]** | | | | | | |
| Absence | Ref | - | Ref | - | Ref | - |
| Presence | -3.21(-7.22,0.81) | 0.12 | 1.04(0.54,2.00) | 0.90 | 0.61(0.32,1.15) | 0.13 |
| **DRIL[e]** | | | | | | |
| Absent | Ref | - | Ref | - | Ref | - |
| Present | -1.50(-5.57,2.57) | 0.47 | 0.92(0.48,1.74) | 0.79 | 0.92(0.49,1.72) | 0.79 |
| **EZ [e]** | | | | | | |
| Intact | Ref | - | Ref | - | Ref | - |
| Not Intact | -9.32(-14.97,-3.66) | *0.001* | 0.46(0.19,1.14) | 0.09 | 0.58(0.23,1.42) | 0.23 |
| Ungradable/Questionable | 0.76(-3.65,5.18) | 0.73 | 1.27(0.64,2.53) | 0.50 | 1.38(0.69,2.77) | 0.37 |
| **ELM [e]** | | | | | | |
| Intact | Ref | - | Ref | - | Ref | - |
| Not Intact | -4.05(-10.95,2.84) | 0.25 | 0.74(0.26,2.12) | 0.58 | 1.13(0.39,3.31) | 0.82 |
| Ungradable/Questionable | 2.86(-1.30,7.01) | 0.18 | 1.45(0.77,2.75) | 0.25 | 1.62(0.85,3.11) | 0.14 |

3 outliers identified and removed from CST and total volume

28 participants with ischemic CRVO at baseline were excluded

[a] For baseline VA, the outcome should be interpreted as the final visual acuity at 52 weeks

[b] Adjusted for baseline VA and treatment arm

[c] Groups 37-54 and <37 was collapsed for outcome 10-letter gainers due to low numbers in group <37 letters that did not improve. Groups 55-70 and >70 were collapsed for outcome >70 letters due to low numbers in group >70 that dropped to <=70 letters by 52 weeks.
[d] Showing only variables that were statistically significant at the 10% threshold (p<0.1).
[e] Adjusted for baseline VA, age, disease duration and treatment arm
Statistically significant p-values at the 5% threshold (p<0.05) have been italicized.
Abbreviations: FP, fractional polynomial; OCT; BCVA, best corrected visual Acuity; CST, Central subfield thickness; SRD, sub-retinal detachment; DRIL, disorganization of retinal inner layers; EZ, ellipsoid zone; ELM, external limiting membrane; AIC, Akaike information criterion; LR, Likelihood-ratio test
