# Supplementary material for: Predictors of Visual Acuity Outcomes after Anti–Vascular Endothelial Growth Factor Treatment for Macular Edema Secondary to Central Retinal Vein Occlusion
Source: Ophthalmol Retina. 2021 Nov;5(11):1115–24. doi: 10.1016/j.oret.2021.02.008 (PMC8565966; doi:10.1016/j.oret.2021.02.008)
Supplement: Table S9 [file mmc17.pdf]

eTable 9. Visual outcome based on various definitions of ischemic CRVO

Different definitions of ischaemia	Baseline BCVA	Final BCVA	Change in BCVA at 100 weeks
Clinician defined ischaemic CRVO in 463 patients – whole LEAVO cohort (n=56)			
Mean (SD)	44(16.3)	63.4(20.9)	20.6(24.4)
Median (IQR)	39(30-60)	69(56-77.5)	22.5(8.5-39)
Patients with RAPD in 267 patients included in this post-hoc analysis (n=25)			
Mean (SD)	42.4(15.1)	66.0(14.3)	24.6(19.4)
Median (IQR)	38.5(28-56)	68(58-77)	22(13-40)
Clinician defined in 267 patients in this post-hoc analysis (n=28)			
Mean (SD)	41.3(15.6)	65.7(17.5)	25.3(20.0)
Media (IQR)	37(28-52)	68.5(61.5-79)	26(12.5-39.5)
>30 disc areas in 106 patients with Optos FA (n=44)			
Mean (SD)	52.8(14.4)	65.8(15.9)	12.8(15.9)
Median (IQR)	55(45.5-62)	72(54-77)	13(5-23)
>50 disc areas in 106 patients with Optos FA (n=20)			
Mean (SD)	48.8(17.0)	63.8(15.3)	15.1(17.0)
Median (IQR)	52.5(33.5-62)	71(50-74.5)	12.5(4.5-23)
>75 disc areas in 106 patients with Optos FA (n=6)			
Mean (SD)	44(16.9)	51.8(15.0)	7.8(16.3)
Median (IQR)	39.5(28-60)	48.5(39-62)	6.5(-1-13)

Abbreviations: BCVA- Best corrected visual acuity; CRVO- central retinal vein occlusion; RAPD- relative afferent pupillary defect; FA- Fluorescein angiography; SD- Standard deviation; IQR- Inter quartile range.
